# Pharmacologic characterization of SHR8443, a novel dual inhibitor of phosphatidylinositol 3-kinase and mammalian target of rapamycin

**DOI:** 10.18632/oncotarget.22439

**Published:** 2017-11-14

**Authors:** Chengying Xie, Xiangling Chen, Mingyue Zheng, Xiaohong Liu, Hongbin Wang, Liguang Lou

**Affiliations:** ^1^ Shanghai Institute of Materia Medica, Chinese Academy of Sciences, Shanghai 201203, China

**Keywords:** BEZ235, drug resistance, mTOR, PI3K, SHR8443

## Abstract

Dysregulation of the phosphatidylinositol 3-kinase (PI3K) pathway occurs frequently in human cancer and contributes to resistance to antitumor therapy. Inhibition of key signaling proteins in this pathway therefore represents an attractive targeting strategy for cancer therapy. Here, we show that SHR8443, an imidazo [4,5-c] quinoline derivative, inhibited mammalian target of rapamycin (mTOR) kinase and PI3K, especially PI3Kα/δ/γ isoforms with picomolar potency, by binding to the ATP subunits of the respective enzymes. Inhibition of PI3K/AKT/mTOR signaling by SHR8443 induced G_1_ phase arrest, autophagy and apoptosis, and resulted in broad anti-proliferative activity against a panel of cancer cells with different genetic backgrounds. Furthermore, SHR8443 overcame resistance to RAF/MEK inhibitors and exhibited synergistic antitumor activity in combination with RAF/MEK inhibitors *in vitro*. Compared with the well-known PI3K/mTOR inhibitor BEZ235, SHR8443 showed broader and stronger efficacy against carcinoma xenografts, including those resistant to anti-HER2 antibody trastuzumab, in association with the inhibition of AKT and S6 phosphorylation in tumor tissues, and also caused no noticeable toxicity. Thus, our preclinical data show that SHR8443 is a dual PI3K/mTOR inhibitor with pharmaceutical properties favorable for use as an anticancer agent.

## INTRODUCTION

Phosphatidylinositol 3-kinases (PI3Ks) are important for regulation of cellular functions, including proliferation, differentiation, metabolism, and migration [1, 2]. PI3Ks activate most of their downstream targets via the serine/threonine kinase, AKT, which phosphorylates downstream substrates such as mammalian target of rapamycin (mTOR), a master regulator of protein translation [3]. The PI3K signaling pathway is essential for tumorigenesis and progression, and is abnormally activated in many types of cancers as a result of gain-of-function mutations in PI3K, amplification of the epidermal growth factor receptor (EGFR) gene, and inactivation of the phosphatase and tensin homolog (PTEN) [4]. Additionally, hyperactivation of the PI3K/AKT pathway could be responsible for resistance to conventional cytotoxic agents and targeted anticancer therapies [5, 6]. Thus, inhibition of this pathway is an attractive anticancer strategy.

Multiple efforts are underway to develop relevant inhibitors of this pathway. Recently, idelalisib (GS-1101, CAL-101), a highly selective PI3Kδ inhibitor, has been approved for the treatment of several hematological malignancies in combination with rituximab. Temsirolimus, an analog of rapamycin that targets mTOR, has been approved for the treatment of advanced kidney cancer [7]. However, because of feedback in the PI3K/AKT/mTOR signaling pathway, inhibition of one output occurs at the expense of activation of the other [8]. Rapamycin monotherapy activates AKT through inhibition of an mTOR-dependent retrograde signal, resulting in decreased antitumor activity [9]. Thus, inhibition of multiple components of the PI3K/mTOR pathway is a logical strategy for improving therapeutic outcomes. In particular, dual inhibition of PI3K and mTOR has been considered an effective strategy for cancer therapy, since it directly targets the most common PI3K mutants and suppresses PI3K-independent activation of mTOR [10]. Dual inhibition of PI3K and mTOR also has the advantage of circumventing resistance to PI3K inhibitors and avoids the compensatory effects of mTOR inhibitors [11]. To date, a number of these agents, including GSK2126458 and PF-04691502, have entered into clinical trials [12–14]. The imidazole [4,5-c] quinoline, which mimics hydrogen bond interactions of the adenine moiety of ATP with the hinge region, has been used as one of the basis for PI3K inhibitors [15], such as NVP-BEZ235 (dactolisib, BEZ235) [16].

**Figure 1:**
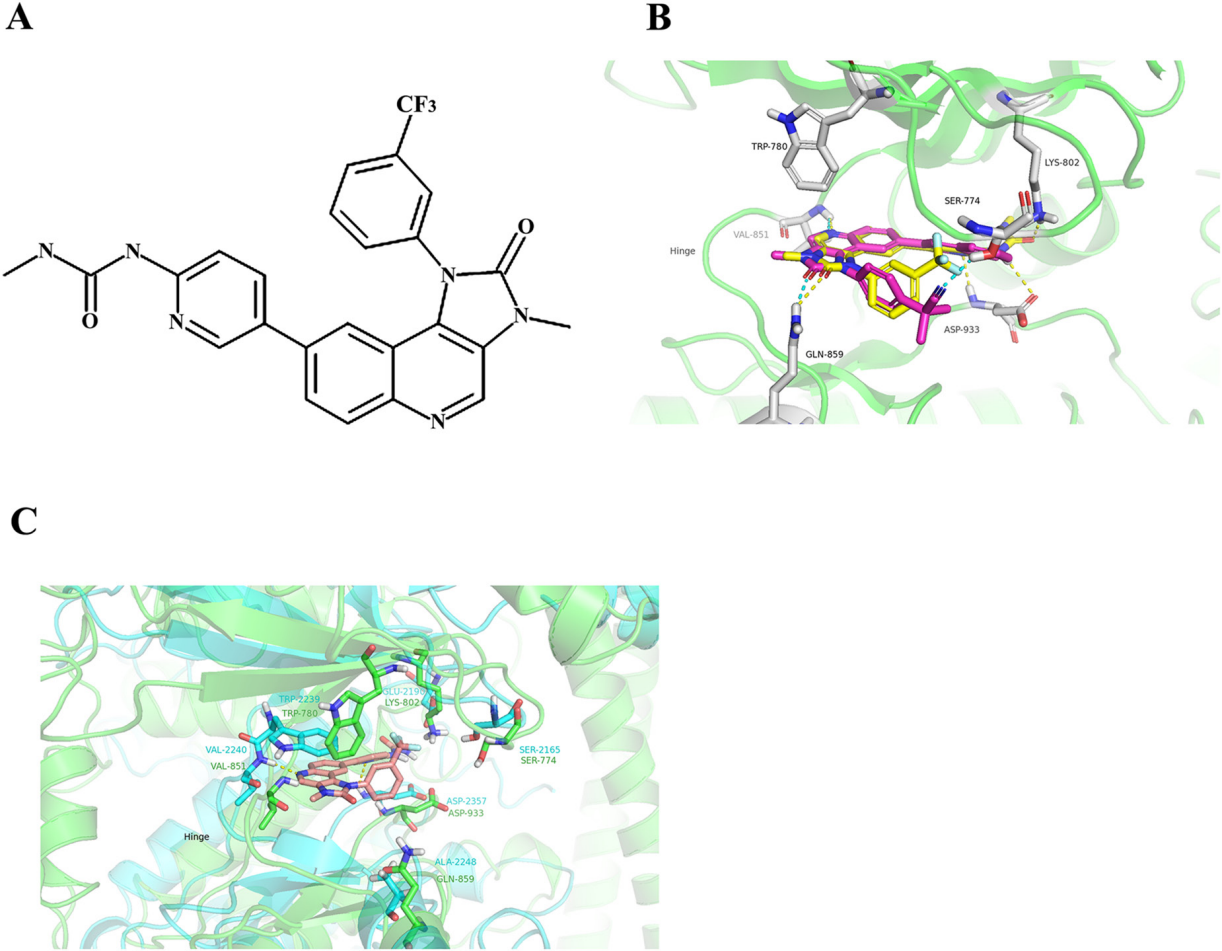
**(A)** Chemical structure of SHR8443. **(B)** The binding modes of BEZ235 and SHR8443 with PI3Kα. The protein was represented as a ribbon diagram (green); SHR8443 (yellow) and BEZ235 (magenta), as well as residues that interacted with these compounds, were shown in stick form. Hydrogen bonds were shown as dashed lines (SHR8443, yellow; BEZ235, cyan) between heavy atoms. **(C)** The binding mode of SHR8443 within mTOR. SHR8443 was represented by wheat-colored sticks; mTOR and PI3Kα were shown as cyan and green ribbon diagrams, respectively. The key residues of mTOR and PI3kα were shown as sticks. Hydrogen bonds were shown as dashed lines (yellow) between heavy atoms.

In this study, we found that SHR8443 (1-methyl-3-(5-(3-methyl-2-oxo-1-(3- (trifluoromethyl)phenyl)-2,3-dihydro-1H–imidazo [4,5-c] quinolin-8-yl) pyridin-2-yl) urea; Figure 1A), a novel imidazole [4,5-c] quinoline derivative, potently and reversibly inhibited the activity of class I PI3K and mTOR kinase. SHR8443 showed broad antitumor activity *in vitro* by blocking inappropriately activated PI3K/mTOR signaling. More importantly, SHR8443 overcame resistance to RAF/MEK inhibitors, and exerted synergistic antitumor activity in combination with RAF/MEK inhibitors. In *in vivo* xenografts, SHR8443 exhibited favorable pharmacologic and pharmaceutical properties without causing noticeable toxicity. Collectively, these results provide a rationale for the ongoing clinical trials of SHR8443.

## RESULTS

### SHR8443 is a potent inhibitor of class I PI3K and mTOR

SHR8443 (Figure 1A), belonging to the class of imidazoquinolines, was tested against PI3Ks in a biochemical kinase assay. As shown in Table 1, IC_50_ values for SHR8443 against p110α, p110δ and p110γ class I PI3K isoforms were 0.1 nM, 0.7 nM and 0.2 nM, respectively. Although the compound showed slightly lower activity against the p110β isoform and mTOR, with IC_50_ values of 12.4 nM and 15.8 nM, respectively, it can be considered as a pan-class PI3K/mTOR inhibitor.

**Table 1:** Enzymatic assays of inhibition of PI3K family members by SHR8443

Kinase	IC_50_ (nM)	
	SHR8443	BEZ235
mTOR	7.8 ± 1.1	28.9 ± 0.0
PI3Kα	0.1 ± 0.0	20.6 ± 4.7
PI3Kβ	15.8 ± 3.8	59.1 ± 23.8
PI3Kδ	0.7 ± 0.2	9.5 ± 7.3
PI3Kγ	0.2 ± 0.2	6.3 ± 0.0

Note: Values are means ± SD (n = 3).

Because most PI3K inhibitors appear to bind to the ATP subunit of PI3K [17], a molecular docking approach was performed to predict the potential binding sites of SHR8443 in PI3Kα and its possible binding mode (Figure 1B); the previously reported PI3K inhibitor, BEZ235, was included as a control [16]. The 2H-imidazo [4,5-c] quinolin-2-one part of both SHR8443 and BEZ235 formed H-bonds with hinge region residues Val851 and Gln859 in PI3Kα; π-π stacking with Trp780 was observed for both molecules. In addition, the cyano group of BEZ235 established a hydrogen bond with Ser774, whereas the trifluoromethyl group of SHR8443 formed dipole-dipole interactions with Ser774. However, the carbamido group of SHR8443 formed two more hydrogen bonds (with Asp933 and Lys802) in the pocket, compared with the quinolyl group of BEZ235.

Next, interactions of SHR8443 with the catalytic site of mTOR were investigated. Although the active sites of PI3Kα and mTOR are significantly different, the key interaction patterns with SHR8443 were similar (Figure 1C). The 2H-imidazo[4,5-c] quinolin-2-one part of SHR8443 formed two hydrogen bonds with the hinge region residues Val851 and Gln859 of PI3Kα, and established one hydrogen bond with residue Val2240 of mTOR. Additional interactions of SHR8443 with mTOR included π-π stacking with Trp2239, dipole-dipole interactions of the trifluoromethyl group of SHR8443 with Ser2165, and hydrogen bond formation of the carbamido group of SHR8443 with Asp2357. These results suggest that SHR8443 inhibits PI3K and mTOR kinase activity by binding to the ATP-binding cleft of these enzymes.

### SHR8443 blocks constitutive PI3K signaling in tumor cells

**Figure 2:**
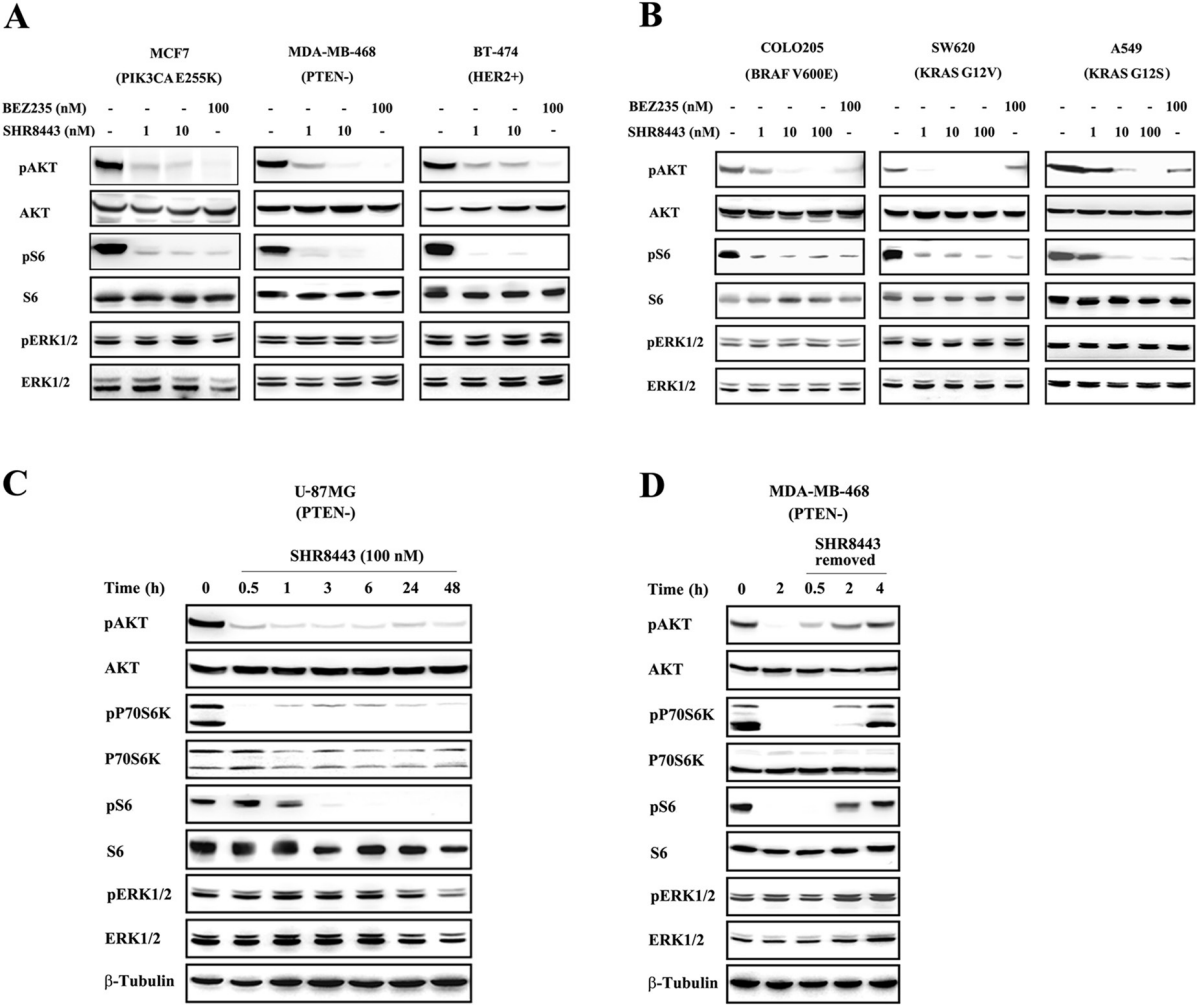
Effects of SHR8443 on PI3K/mTOR signaling in cancer cell lines with different genetic backgrounds **(A)** Breast cancer cell lines (MCF7, BT-474 and MDA-MB-468) and **(B)** KRAS or BRAF mutated cell lines (COLO205, SW620 and A549) were treated with increasing concentrations of SHR8443 or 100 nM BEZ235 for 3 h. **(C)** U-87MG cells were treated with 100 nM SHR8443 for the indicated time. **(D)** Reversibility of PI3K/mTOR signaling. MDA-MB-468 cells were treated with 100 nM SHR8443 for 2 h, with or without further culture in drug-free medium for the indicated time after washing with PBS. Whole-cell lysates were analyzed by Western blotting using the indicated antibodies.

To investigate whether the enzyme inhibitory properties of SHR8443 translate into modulation of the PI3K/mTOR signaling pathway, we assessed the phosphorylation status of downstream substrates in tumor cell lines with different genetic backgrounds. First, the effects of SHR8443 on breast cancer cells, including MCF7 (mutated p110α), MDA-MB-468 (loss of PTEN), and BT-474 (HER2+) were examined. As shown in Figure 2A, SHR8443 inhibited both AKT and S6 phosphorylation in these breast cancer cell lines.

Next, we investigated whether a constitutively activated RAS/MAPK pathway interfered with SHR8443-mediated inhibition of PI3K/mTOR signaling by examining phosphorylation of downstream targets in cell lines in which the MAPK pathway is activated. SHR8443 significantly inhibited the phosphorylation of AKT and S6 in the COLO205 cell line harboring a mutant form of the proto-oncogene BRAF, and A549 and SW620 cell lines harboring a mutant form of the proto-oncogene KRAS (Figure 2B). No change in the level of phosphorylated extracellular signal-regulated kinase (pERK) was observed in any of these cell lines after treatment with SHR8443.

Time course experiments further showed that SHR8443 (100 nM) treatment of U-87MG glioma cells (loss of PTEN) rapidly inhibited phosphorylation of AKT, S6K and S6; moreover, this inhibitory effect lasted at least 48 h (Figure 2C). Similar effects were observed in MDA-MB-468 cells, which also lack PTEN expression. Notably, removal of SHR8443 from the medium after treatment of MDA-MB-468 cells was followed by an equally rapid recovery of these downstream effectors, suggesting that the inhibitory activity of SHR8443 is reversible (Figure 2D).

### SHR8443 exhibits broad antitumor activity *in vitro*

**Figure 3:**
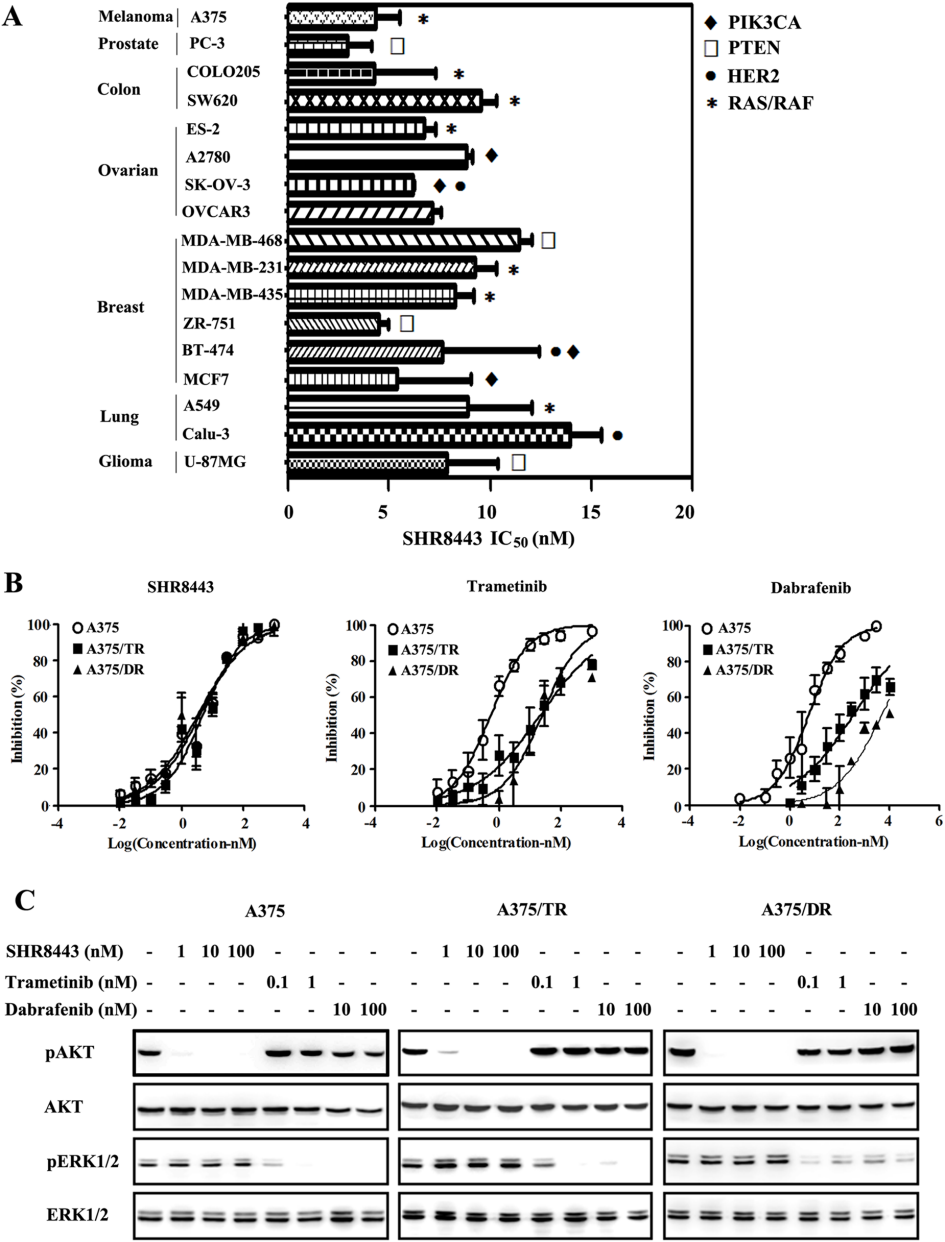
Cell profile of SHR8443 cytotoxicity **(A)** Anti-proliferative activity of SHR8443 against a panel of cancer cell lines with different tissues of origin and genetic backgrounds. Tumor cell lines harboring a PIK3CA mutation (♦), HER2 amplification (●), PTEN deficiency (□), or RAS/RAF mutation (^*^) were treated with different concentrations of SHR8443 for 72 h. Cell viability was determined by SRB assay. IC_50_ values were presented as means ± SD of three independent experiments. **(B)** Melanoma cell lines A375, A375/TR (trametinib-resistant), and A375/DR (dabrafenib-resistant) were treated with different concentrations of SHR8443, dabrafenib, or trametinib for 72 h. Cell viability was determined by SRB assay. **(C)** Melanoma cell lines A375, A375/TR and A375/DR were treated with SHR8443, dabrafenib or trametinib for 2 h. Whole-cell lysates were analyzed by Western blotting using the indicated antibodies.

Because the PI3K/mTOR signaling pathway regulates important cellular functions, including cell proliferation, we investigated the growth-inhibitory effects of SHR8443 against a panel of human tumor cell lines. SHR8443 inhibited the proliferation of all tested tumor cells regardless of their tissue of origin and genetic abnormalities, exhibiting IC_50_ values ranging from 2.9 to 13.9 nM (average, 7.5 nM; Figure 3A). No significant tissue specificity was observed after treatment with SHR8443 in these cell lines.

Furthermore, SHR8443 treatment effectively blocked PI3K/AKT activation and exerted similar cytotoxic effects on trametinib-resistant A375/TR and dabrafenib-resistant A375/DR cells as the parental A375 cells. The IC_50_ values were 4.9 nM, 3.4 nM and 4.1 nM for A375/TR, A375/DR and A375 cells, respectively (Figure 3B). Although both trametinib and dabrafenib blocked ERK1/2 activation in resistant cells (Figure 3C), neither RAF/MEK inhibitor was able to effectively block cell proliferation (Figure 3B), suggesting that the resistant cells were no longer RAF/MEK dependent. The resistance factor (RF) for trametinib was 39.8 and 45.1 in A375/TR and A375/DR cell lines; the corresponding values for dabrafenib in these two resistant cell lines were 61.0 and 839.2. Taken together, these data indicate that SHR8443 shows a broad range of antitumor activity *in vitro* and is capable of overcoming resistance to RAF/MEK inhibitors.

### SHR8443 causes cell cycle arrest, autophagy, and apoptosis

**Figure 4:**
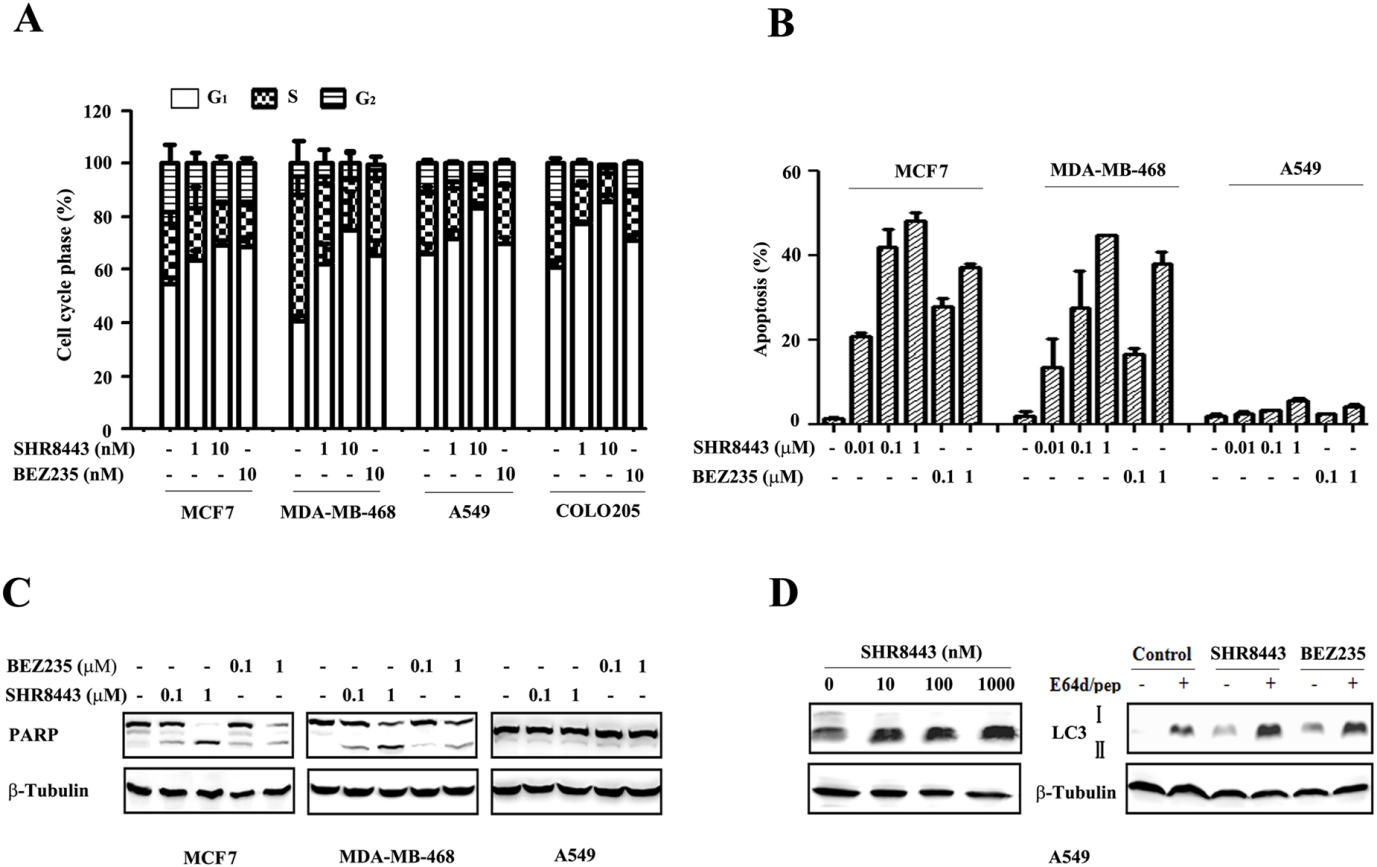
SHR8443 causes cell cycle arrest, autophagy, and apoptosis **(A)** Cell-cycle phase histograms of MCF7, MDA-MB-468, COLO205 and A549 cell lines following treatment with SHR8443 or BEZ235 at the indicated concentration for 24 h. **(B)** MCF7, MDA-MB-468 and A549 cells were treated with SHR8443 or BEZ235 at the indicated concentrations for 72 h, and then analyzed by annexin V-FITC/PI staining and flow cytometry. **(C)** After treatment of cells with SHR8443 or BEZ235 for 72 h, whole-cell lysates were immunoblotted with an anti-PARP antibody. **(D)** A549 cells were treated with SHR8443 (left), BEZ235, or the combination of SHR8443/BEZ235 (100 nM) with E64d/pep (10 mg/mL) for 48 h. Whole-cell lysates were analyzed by immunoblotting with an anti-LC3 antibody.

To analyze the mechanism of cytotoxicity, we next examined the effects of SHR8443 on the cell cycle profile. Treatment with SHR8443 for 24 h induced a concentration-dependent G1-phase cell-cycle arrest in MCF7, MDA-MB-468, COLO205, and A549 cell lines (Figure 4A). Notably, this effect of SHR8443 was independent of the genetic backgrounds of tested tumor cells. Our results also showed that KRAS- and BRAF-mutant containing A549 and COLO205 cell lines, respectively, were less sensitive to BEZ235, consistent with a previous report [10].

To better understand the function of PI3K in human tumor cells, we measured apoptosis induced by SHR8443 using annexin V-FITC/PI staining and FACS analysis. These experiments demonstrated that SHR8443 induced a concentration-dependent increase in necrotic/apoptotic cell death in both MCF7 and MDA-MB-468 cells, but not in A549 cells (Figure 4B). The induction of apoptosis by SHR8443 was further evidenced by cleavage of PARP in both MCF7 and MDA-MB-468 cells. Consistent with FACS analysis results, there was no detectable cleaved PARP in A549 cells, even at an SHR8443 concentration of 1 μM (Figure 4C). These results suggest that PI3K/mTOR inhibitors induce tumor cell apoptosis in a cell-type–dependent manner.

Previous studies have shown that inhibition of the PI3K/mTOR pathway induces autophagy, a type II programmed cell death [18]. To assess this, we examined LC3 protein, a hallmark of cells undergoing autophagy [19]. SHR8443 treatment increased the production of LC3-II in a concentration-dependent manner and exhibited further enhanced activity when combined with the protease inhibitor E64d/pep (Figure 4D). Taken together, these findings indicate that SHR8443 induces cell cycle arrest and autophagy in KRAS mutant A549 cells.

### Combined treatment SHR8443 and BRAF/MEK inhibitors enhances antitumor activity in BRAF mutant cells

**Figure 5:**
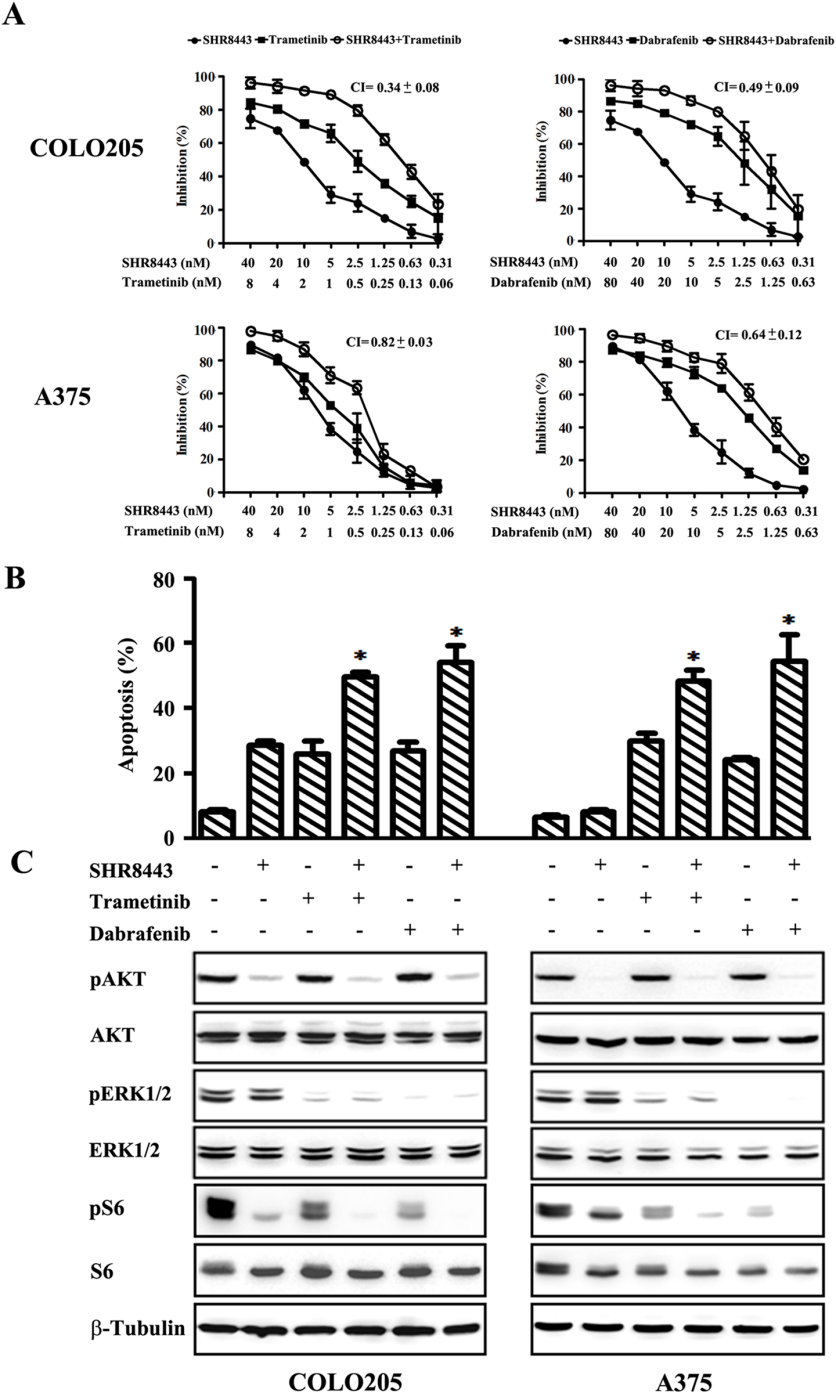
Combined treatment of BRAF mutant A375 and COLO205 cells with SHR8443 and dabrafenib or trametinib **(A)** A375 and COLO205 cells were incubated with the indicated concentrations of each compound or their combinations for 72 h. Cell viability was determined by SRB assay. CI, combination index. **(B)** A375 and COLO205 cells were incubated with SHR8443 (100 nM), trametinib (10 nM), dabrafenib (100 nM), or their combinations for 72 h, and analyzed by annexin V-FITC/PI staining and flow cytometry. ^*^P < 0.05, versus either single agent treatment. **(C)** A375 and COLO205 cells were incubated with SHR8443 (100 nM), trametinib (10 nM), dabrafenib (100 nM), or their combinations for 2 h. Whole-cell lysates were analyzed by Western blotting using the indicated antibodies.

Combinations of PI3K inhibitors with antitumor drugs produce higher response rates than single-agent treatments [20, 21]. In light of this, we evaluated the effects of combining SHR8443 with RAF/MEK inhibitors on the proliferation of BRAF mutant COLO205 and A375 cell lines. SHR8443 combined with trametinib or dabrafenib exerted synergistic cytotoxicity in both cell lines (Figure 5A). The combination index (CI) values for SHR8443 combined with trametinib were 0.34 in COLO205 cells and 0.82 in A375 cells; the corresponding CI values for SHR8443 combined with dabrafenib were 0.49 and 0.64.

Next, effects of combined treatment with SHR8443 and RAF/MEK inhibitors on apoptosis in BRAF mutant cells were investigated. In COLO205 cells, SHR8443, trametinib and dabrafenib individually induced apoptosis, and the combination of SHR8443 and trametinib/dabrafenib showed enhanced apoptosis induction (Figure 5B). Although SHR8443 alone did not induce noticeable apoptosis in A375 cells, it enhanced the apoptosis induced by trametinib or dabrafenib (Figure 5B).

To gain insight into the mechanisms by which the combination of SHR8443 and RAF/MEK inhibitors exerts enhanced anticancer activity, we analyzed the effects of the combination on PI3K/mTOR signaling compared with either agent alone. SHR8443 combined with dabrafenib or trametinib simultaneously inhibited the phosphorylation of AKT and ERK in both COLO205 and A375 cells (Figure 5C). Importantly, these combined treatment regimens showed augmented inhibitory effects on S6 phosphorylation in both cell lines compared with each single agent alone.

### *In vivo* antitumor activity of SHR8443

**Figure 6:**
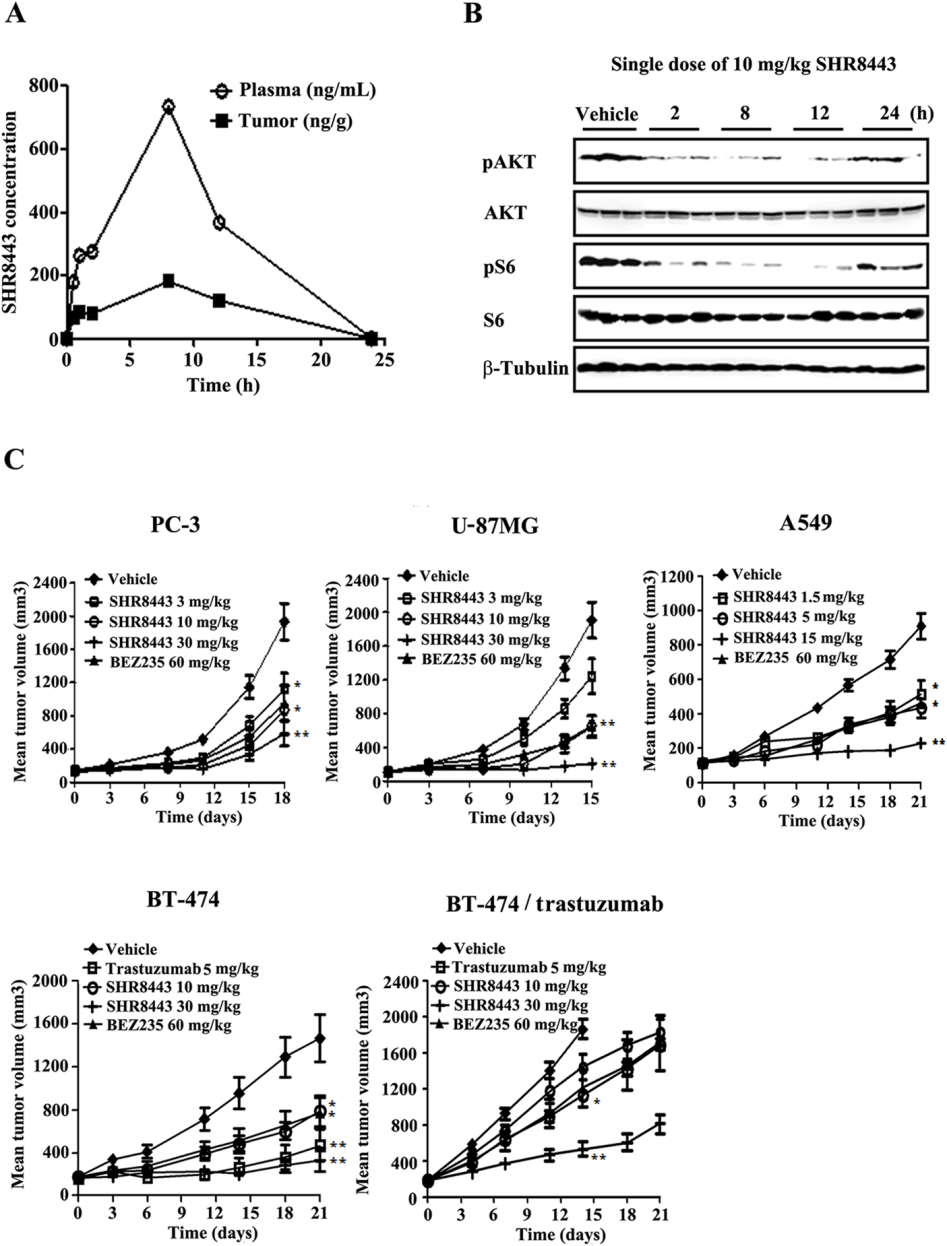
*In vivo* study of SHR8443 in mouse xenograft models **(A, B)** Pharmacokinetic/pharmacodynamic studies of SHR8443. U-87MG tumor-bearing mice were orally administered a single 10-mg/kg dose of SHR8443 and sacrificed at the indicated times. The concentration of SHR8443 in blood plasma and tumor tissues was determined (A). In parallel, tumor extracts were analyzed by Western blotting (B). **(C)** Antitumor activity of SHR8443 against xenografts. U-87MG, PC-3, A549, BT-474 and BT-474/trastuzumab tumor-bearing mice were orally administered BEZ235 or SHR8443 daily, or intravenously injected with trastuzumab twice a week. Tumor volumes were measured twice a week. ^*^P < 0.05, ^**^P < 0.01 versus vehicle.

Given the encouraging activity of SHR8443 *in vitro*, we next analyzed its pharmacokinetic/pharmacodynamic profiles in the U-87MG xenograft model. As shown in Figure 6A, following treatment at a dose of 10 mg/kg, SHR8443 appeared in plasma and tumor tissue with C_max_ values of 735 ng/mL and 181 ng/g, respectively at 8 h. The inhibitory effects of SHR8443 on pAKT and pS6 levels in tumor tissue were concordant with the observed temporal changes in plasma and intratumoral concentrations of SHR8443 over a 24-h period (Figure 6A, 6B).

The antitumor activity of SHR8443 was investigated against a panel of tumor xenografts with different genetic backgrounds. Oral administration of SHR8443 significantly inhibited the growth of all tested human tumor xenografts in a dose-dependent manner, and showed stronger *in vivo* antitumor activity than BEZ235 (Figure 6C). SHR8443 at a dose of 30 mg/kg led to significant tumor growth inhibition (TGI) of 87%, 75% and 95% in BT-474, PC-3 and U-87MG xenografts, respectively, on the final treatment day. Moreover, tumor regression was observed in one of six tumors at a dose of 30 mg/kg in both BT-474 and U-87MG xenografts. SHR8443 at a dose of 15 mg/kg showed a TGI of 86% in KRAS mutant A549 xenografts. At a dose of 30 mg/kg, it also exhibited significant antitumor activity in trastuzumab-resistant BT-474/trastuzumab xenografts with a TGI value of 79%, compared with 39% for 60 mg/kg BEZ235. In comparison, TGI values for 5 mg/kg of trastuzumab in BT-474/trastuzumab and parent BT-474 xenografts were 25% and 80%, respectively. All these treatments were well tolerated, as evidenced by the absence of drug-related deaths and significant body weight loss in all groups during the course of the experiment (Supplementary Figure 1).

## DISCUSSION

The PI3K/mTOR signaling pathway is one of the most frequently disrupted intracellular pathways in human cancer, where it contributes significantly to tumor progression and development of resistance to chemotherapeutic drugs [4, 5]. Over the past 20 years, medicinal chemistry activities have been directed toward the development of pan-PI3K inhibitors with improved pharmacological characteristics [22, 23]. Here, we demonstrated that SHR8443, a novel orally available inhibitor of PI3K/mTOR signaling, exhibited broad antitumor activity against human cancer cell lines independent of their genetic backgrounds both *in vitro* and *in vivo*, notably overcoming resistance to targeted anticancer therapies.

The imidazole [4,5-c] quinoline is usually used as one of the basis for PI3K inhibitors, such as the broad-spectrum PI3K/mTOR inhibitor BEZ235 [15, 16]. Molecular modeling showed that SHR8443, a novel [4,5-c]quinoline derivative, bound to the ATP-binding pocket of PI3K and mTOR. However, the trifluoromethyl group of SHR8443 formed dipole-dipole interactions with Ser774 of PI3K, whereas the cyano group of BEZ235 established a hydrogen bond with this same residue. Importantly, the carbamido group of SHR8443 formed two additional hydrogen bonds (with Asp933 and Lys802) in the pocket within PI3K compared with the quinolyl group of BEZ235. In mTOR, SHR8443 only established one hydrogen bond (with Asp2357). These extra H-bond interactions with PI3K may account for the higher selectivity of SHR8443 for PI3Kα relative to mTOR and its greater inhibitory activity toward PI3Kα compared with BEZ235. Indeed, SHR8443 showed improved inhibitory activity toward PI3Kα/δ/γ, with picomolar IC_50_ values in enzyme assays. SHR8443 showed slightly lower, but still significant, inhibitory activity against the p110β isoform and mTOR at the nanomolar level. As previously reported, SHR8443, similar to BEZ235, had little inhibitory activity against a panel of other protein kinases at a concentration of 1000 nM (data not shown). The specific targeted inhibition of PI3K/mTOR, together with high potent for PI3Kα, by SHR8443, may imply a favorable therapeutic window and result in minimal side-effects in clinic.

It is well known that the PI3K/mTOR signaling pathway is often modulated by other signaling elements, such as amplification of HER2, loss of PTEN, and mutation in PI3K [4]. In this study, we found no correlation between the biochemical activity of SHR8443 and the basal activity of the PI3K/AKT pathway. However, SHR8443 inhibited the phosphorylation of PI3K and mTOR downstream effectors in tumor cell lines harboring different mutations that modulated PI3K/AKT activity. Moreover, the fact that p70S6K, the upstream kinase of S6 ribosomal protein, is sequentially activated by both mTORC1 and PDK1 may explain why S6 phosphorylation is more sensitive to inhibition by SHR8443 than AKT, which is activated only by mTORC2 [10, 24]. Thus, the inhibitory activity toward p70S6K and S6 may be an additive effect reflecting the inhibition of its two major activators by SHR8443. Furthermore, upregulation of the RAS/MAPK pathway was not detected after SHR8443 treatment, as it simultaneously inhibited PI3K and mTORC1/2 [25]. Notably, the targeting inhibitory activity by SHR8443 was independent of the genetic background of the cancer cells, indicating that neither PTEN loss nor the mutation status of upstream tyrosine kinases necessarily predicts inhibition of these events.

SHR8443 inhibited proliferation in all tested tumor cells, independent of their tissue of origin or genetic background, with a mean IC_50_ value of 7.5 nM. A previous study showed that tumor cells harboring either KRAS/BRAF mutations or EGFR amplification were slightly less sensitive to BEZ235 than other cells [10]. Here, we found no significant connection between genetic background and sensitivity to SHR8443, which was evidenced by a mean IC_50_ value of 7.3 nM in KRAS/BRAF mutant tumor cells. Cross-resistance to BRAF-, MEK1/2- and PI3K/mTOR-specific inhibitors in BRAF mutant melanoma cells has been demonstrated [26]. Here, both trametinib-resistant A375/TR and dabrafenib-resistant A375/DR resistant cells also displayed cross-resistance. For example, the A375/TR cell line was less sensitive to dabrafenib than the parental line, with the RF of 61.0; the A375/DR cell line was also less sensitive to trametinib, with the RF of 45.1. Activation of the PI3K pathway has been shown to induce resistance to chemotherapy, whereas inhibition of this pathway restores sensitivity [27, 28]. In agreement with this, we found that SHR8443 alone significantly inhibited PI3K/AKT signaling and cell proliferation in both the parent and resistant cell lines with the same potency.

Although both SHR8443 and BEZ235 induced cell cycle arrest in G_1_, the A549 (KRAS mutation) and COLO205 (BRAF mutation) cell lines were slightly less sensitive to BEZ235, whereas all tested cells showed similar sensitivity to SHR8443. Previous studies reported that induction of G_1_ arrest, but not apoptosis, following treatment of U-87MG, PC3M and sarcoma cell lines with BEZ235 [16, 29], the PI3K inhibitor ZSTK474 [30], or the PI3K/mTOR inhibitor PI103 [31]. BEZ235 was shown to selectively induce apoptosis in HER2-amplified and/or PIK3CA mutant breast cancer cells through inhibition of PI3K/AKT/mTORC2 [32]. In agreement with this, we found that SHR8443 treatment induced apoptosis in the breast cancer cell lines, MCF7 and MDA-MB-468, but not in the A549 cell line. Overall, SHR8443 effectively permeated cells to modulate signaling pathways downstream of PI3K/mTOR independent of mutations related to PI3K/AKT/mTOR and RAF/RAS/MEK pathways and displayed broad antitumor activity. The mechanism underlying this sensitivity to SHR8443 is currently unclear and warrants further investigation.

Previous studies support the hypothesis that the combination of PI3K inhibitors and MEK inhibitors prevents potential feedback effects and produces higher response rates than single-agent treatments [14, 20, 33, 34]. Although there was no concomitant activation of ERK by SHR8443, the combination of SHR8443 and trametinib/dabrafenib showed synergistic cytotoxicity in BRAF mutant COLO205 and A375 cells, reflecting dual inhibition of PI3K/AKT and MEK/ERK signaling. Interestingly, combination treatment caused stronger inhibition of S6 phosphorylation compared with either drug alone. These results suggest that SHR8443 alone or in combination with BRAF/MEK inhibitor may be a therapeutic option for resistant and BRAF mutant tumors.

SHR8443 showed good pharmacokinetic properties and effectively inhibited mTOR as well as PI3K signaling in tumor tissue after once daily oral dosing. It has been shown that PI3K/mTOR signaling is upregulated in trastuzumab-resistant breast cancer cells, and blockade of this pathway restores sensitivity toward trastuzumab [35]. The antitumor activity of SHR8443 *in vivo* using xenograft models with different oncogenic mutations showed no correlation between tumor mutations and response to SHR8443. SHR8443 was well tolerated when administered orally and displayed broader and stronger antitumor activity than BEZ235 against tumor xenografts formed from PTEN-null U-87MG and PC-3 cells, KRAS-mutated A549 cells, PI3K-mutated BT-474 cells, and trastuzumab-resistant BT-474/trastuzumab cells. Taken together, our data indicate that SHR8443 is a good alternative option for anticancer therapy, including cases with a mutation profile and resistance to targeted anticancer therapies.

In summary, SHR8443 is a novel, orally available, dual PI3K and mTOR inhibitor. The favorable pharmacokinetic and pharmacodynamic properties of SHR8443, and its resulting efficacy against a wide range of tumors, provide a compelling rationale for the clinical evaluation of this drug. In particular, it may be expected that SHR8443 would also be effective in some of cancer patients with acquired resistances to targeted therapies. The fact that SHR8443 is currently undergoing phase I clinical trials in China reinforces this conclusion.

## MATERIALS AND METHODS

### Materials

SHR8443, provided by Jiangsu Hengrui Medicine Co. Ltd. (Jiangsu, China), was prepared as 10 mM stock solutions in dimethylsulfoxide for *in vitro* studies or normal saline for *in vivo* studies. BEZ235, dabrafenib, and trametinib were purchased from Selleckchem (Houston, TX, USA). Trastuzumab was purchased from Roche (Basel, Switzerland). E64d/pep was purchased from Sigma-Aldrich (St. Louis, MO, USA).

Appropriate primary antibodies to poly (ADP-ribose) polymerase (PARP), microtubule-associated protein-1 light chain 3 (LC3), AKT, pS6^235/6^, S6, pP70S6K^T389^, P70S6K, pAKT^S473^ and pERK1/2 were purchased from Cell Signaling (Beverly, MA, USA). The ERK1/2 antibody was purchased from Santa Cruz Biotechnology (Santa Cruz, CA, USA).

### PI3K/mTOR ELISAs

PI3K enzyme-linked immunosorbent assays (ELISAs) were performed using the PI3Kinase Activity/Inhibitor Assay Kit (Millipore, Bedford, MA, USA) according to the manufacturer's instructions as described previously [36]. mTOR kinase activity was assayed using the K-LISA mTOR Activity Kit (Calbiochem, Bedford, MA, USA) according to the manufacturer's instructions.

### Molecular modeling of the SHR8443-protein complex

The crystal structures of PI3Kα (Protein Data Bank [PDB] Code: 4JPS) [37] and mTOR (PDB Code: 4JSX) [38] were obtained as target structures for molecular docking simulations. Molecular modeling calculations were performed using Schrödinger software (release 2015-2). Three-dimensional structures of SHR8443 and BEZ235 were generated with the Maestro module [39] and prepared using the LigPrep [40] module in the Schrödinger package. Docking studies were performed using the Glide module [41] in Schrödinger with default parameters, unless otherwise noted. The obtained docked poses were analyzed with Maestro [39] and PyMOL [42].

### Cell culture

The following human tumor cell lines were obtained from the American Type Culture Collection (Manassas, VA, USA): breast tumor cell lines, MDA-MB-231, MDA-MB-435, MDA-MB-468, BT-474 and MCF7; lung tumor cell lines, A549 and Calu-3; ovarian tumor cell lines, SK-OV-3 and OVCAR3; colon tumor cell lines, SW620 and COLO205; glioma cell line, U-87MG; prostate tumor cell line, PC-3; and melanoma cell line, A375. All cell lines were cultured according to instructions provided by the American Type Culture Collection. The ovarian tumor cell line A2780 was obtained from the National Cancer Institute (Bethesda, MD, USA) and cultured according to the instructions provided. The dabrafenib- and trametinib-resistant A375 cell lines, A375/DR and A375/TR, respectively, were established by culturing parental cells in medium containing gradually increasing concentrations of dabrafenib (0.1–5,000 nM) or trametinib (0.05–50 nM) over the course of 12 months. The trastuzumab-resistant BT-474 cell line, BT-474/trastuzumab, was established by initially culturing in medium containing 100 ng/mL trastuzumab and gradually increasing drug concentration to 10 μg/mL. All the cells have been tested and authenticated by Genesky Biotechnologies. Inc. (Shanghai, China) using fluorescent amplified restriction fragment polymorphism (FAFLP) method.

### Cell proliferation assay

Tumor cells were seeded in 96-well plates and treated with serial dilutions of drugs alone or as a combination of two drugs. After a 72-h incubation, cell proliferation was evaluated by sulforhodamine B (SRB; Sigma) assay [43]. The potency of drugs in inhibiting cell proliferation was expressed as 50% inhibitory concentration (IC_50_) values, determined using GraphPad Prism version 5 curve-fitting software (GraphPad Software, San Diego, CA, USA). The CI value which defines the interaction between two drugs as synergistic (CI < 0.95), additive (CI = 0.95–1.05) or antagonistic (CI > 1.05), was determined on the basis of the median-effect principle using CalcuSyn software.

### Flow cytometry analysis

Cells were harvested and fixed in ice-cold 70% ethanol at −20°C overnight. Fixed cells were then treated with 50 μg/mL of propidium iodide and RNase A at 37°C for 30 min. The cell cycle distribution was measured using a FACScan flow cytometer (BD Biosciences, San Jose, CA, USA) and analyzed with ModFit LT Mac V3.0 software. Apoptosis was measured using a FITC Annexin V Apoptosis Detection Kit (BD Biosciences) according to the manufacturer's instructions. Fluorescence was acquired with an Accuri C6 Plus instrument (BD Biosciences).

### Western blotting

After drug treatment, cells were lysed in sodium dodecyl sulfate (SDS) sample buffer (100 mM Tris-HCl pH 6.8, 2% SDS, 20% glycerol, 1 mM dithiothreitol). Cell lysates containing equal amounts of protein were resolved by SDS-PAGE and transferred to polyvinylidene difluoride membranes. Blots were probed with primary antibodies, and then incubated with the appropriate secondary antibodies (Millipore). Immunoreactive bands were visualized using enhanced chemiluminescence reagents (Millipore).

### *In vivo* study

Female, 5–6-wk-old Balb/cA-nude mice were purchased from Shanghai Laboratory Animal Center, Chinese Academy of Sciences (Shanghai, China). Tumor xenografts of human BT-474, BT-474/trastuzumab, PC-3, U-87MG, or A549 cells were established by subcutaneously inoculating cells into nude mice. Once tumor volumes reached ~100–200 mm^3^, mice were randomly assigned to control (n = 12) or treatment (n = 6) groups, and treated with vehicle, BEZ235, SHR8443, or trastuzumab. Tumor volume was calculated as (length × width^2^)/2.

Pharmacokinetic/pharmacodynamic studies were carried out as described previously [36]. Mice bearing U-87MG tumors received a single oral dose of 10 mg/kg SHR8443 or vehicle, and tumor tissues and blood were collected at different times. Concentrations of SHR8443 in plasma and tissue were determined by HPLC/tandem mass spectrometry. Tumor samples were analyzed by Western blotting. Animal experiments were conducted in accordance with the Institutional Animal Care and Use Committee guidelines of Shanghai Institute of *Materia Medica*, Chinese Academy of Sciences.

### Data analysis

Data are presented as means ± standard errors of the mean (SEM) and were plotted using GraphPad Prism Version 5. An unpaired two-tailed Student's t-test was used to test for significance, where indicated. Differences were considered significant at *P* < 0.05.

## SUPPLEMENTARY MATERIALS FIGURE



## Abbreviations

PI3K: phosphatidylinositol 3-kinase
ERK: extracellular signal-regulated protein kinase
mTOR: mammalian target of rapamycin
EGFR: epidermal growth factor receptor gene
ELISA: enzyme-linked immunosorbent assay
SRB: sulforhodamine B
SDS: sodium dodecyl sulphate
IC_50_: half-maximal inhibitory concentration
A375/DR: dabrafenib-resistant A375
A375/TR: trametinib-resistant A375
RF: resistance factor
CI: combination index
TGI: tumor growth inhibition
MAPK: mitogen-activated protein kinase.
