# Supplementary material for: Pharmacologic characterization of SHR8443, a novel dual inhibitor of phosphatidylinositol 3-kinase and mammalian target of rapamycin
Source: Oncotarget. 2017 Nov 14;8(64):107977–90. doi: 10.18632/oncotarget.22439 (PMC5746119; doi:10.18632/oncotarget.22439)
Supplement: Supplementary file 1 [file oncotarget-08-107977-s001.pdf]

Pharmacologic characterization of SHR8443, a novel dual inhibitor of phosphatidylinositol 3-kinase and mammalian target of rapamycin

SUPPLEMENTARY MATERIALS

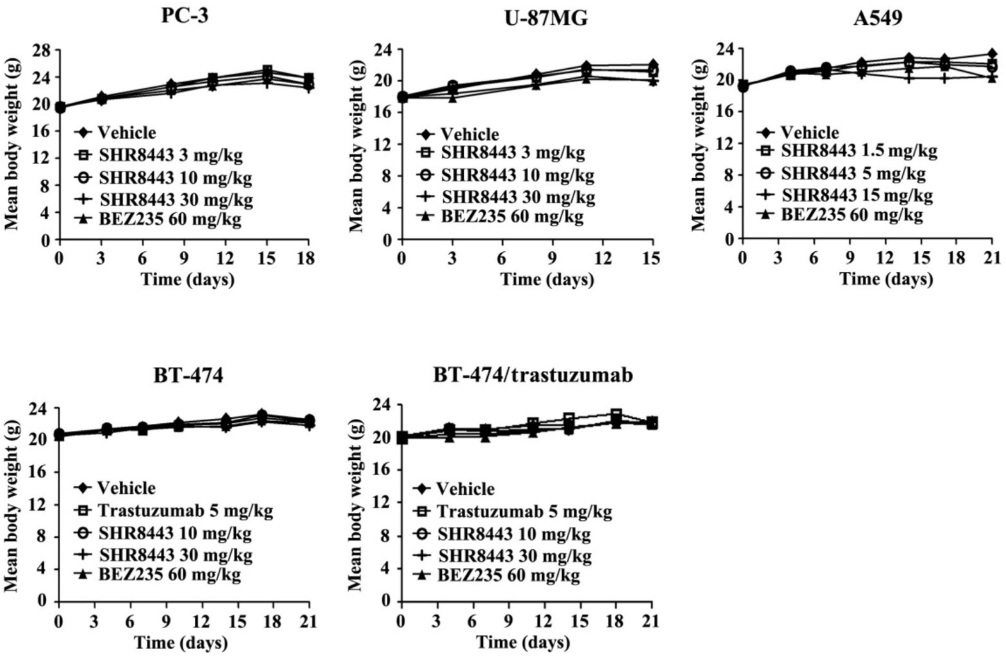

Supplementary Figure 1: *In vivo* study of SHR8443 in mouse xenograft models. U-87MG, PC-3, A549, BT-474 and BT-474/trastuzumab tumor-bearing mice were orally administered with BEZ235 or SHR8443 daily, or intravenously injected with trastuzumab twice a week. Body weight was determined twice a week.
